# Semantic Relations in a Categorical Verbal Fluency Test: An Exploratory Investigation in Mild Cognitive Impairment

**DOI:** 10.3389/fpsyg.2019.02797

**Published:** 2019-12-17

**Authors:** Davide Quaranta, Chiara Piccininni, Alessia Caprara, Alessia Malandrino, Guido Gainotti, Camillo Marra

**Affiliations:** ^1^Area of Neuroscience, Fondazione Policlinico Universitario A. Gemelli IRCCS, Rome, Italy; ^2^Institute of Neurology, Catholic University of the Sacred Heart, Rome, Italy; ^3^Department of Clinical and Behavioral Neurology, IRCCS Fondazione Santa Lucia, Rome, Italy

**Keywords:** semantic memory, dementia of the Alzheimer type, mild cognitive impairment, category fluency task, semantic proximity

## Abstract

Categorical verbal fluency tests (CFT) are commonly used to assess the integrity of semantic memory in individuals with brain damage. Persons with Dementia of the Alzheimer’s Type display a reduced output on CFT, and a similar pattern has been reported in persons with amnesic Mild Cognitive Impairment (aMCI). The aims of the present study were to assess whether the semantic relations between lexical entries produced on a categorical fluency test were different between healthy persons and those with aMCI, and whether this difference was more pronounced in individuals who converted to dementia during a 3-year follow-up period.

**Methods:**

We recruited 34 individuals with aMCI and 29 matched healthy persons. During the follow-up period, 10 individuals converted to Dementia (aMCI-conv). Two measures assessing semantic relations between consecutively produced word pairs (Path length and Extended Gloss Overlap) were obtained from the Wordnet database.

**Results:**

The number of word pairs analyzed among the healthy participants (HP) and persons with aMCI were 498 (birds: 262; pieces of furniture: 236) and 395 (birds: 174; pieces of furniture: 221), respectively. Path length was lower in aMCI-conv than in HP (*p* = 0.035), but no differences were found between stable aMCI and HP, and between aMCI-stable and aMCI-conv. The ANOVA for lexical entries belonging to the “birds” category showed a significant effect of group (*F* = 5.630; *p* = 0.004); the *post hoc* analysis showed a significant difference between HP and aMCI-conv (*p* = 0.003). The “pieces of furniture” category was significantly affected by group (*F* = 4.107; *p* = 0.017); the *post hoc* test showed significant differences between aMCI-conv and healthy individuals (*p* = 0.049), and between aMCI-conv and stable aMCI (*p* = 0.001).

**Discussion:**

Individuals with aMCI who convert to dementia show a deterioration in the semantic relations between lexical entries, produced on a CFT. This phenomenon may be interpreted as a marker of a very early disruption of semantic memory.

## Introduction

Verbal fluency tasks are traditionally used in the assessment of patients with dementia, particularly with Alzheimer’s disease. The most common versions of verbal fluency tests consist of the production of words in a limited amount of time starting with a given letter (phonological verbal fluency tasks) or within a semantic category (categorical verbal fluency tasks) (Strauss et al., 2006; Lezak, 2012). Both tasks produce the same kind of score (the number of words per unit of time), but are based on quite different mechanisms. Categorical verbal fluency tests (CFT) require the retrieval of the content of semantic memory, in which concepts and words are hierarchically organized whereas phonological verbal fluency tasks demand an effortful exploration of the lexical system and require the intervention of an unusual strategy based on phonological, rather than semantic activation (Robinson et al., 2012; Vita et al., 2014). CFT are considered a reliable tool to assess the integrity of semantic memory.

Previous studies have reported impaired performance in CFT in Dementia of the Alzheimer’s Type (DAT) (Monsch et al., 1992; Henry et al., 2004) and in Mild Cognitive impairment (MCI) (Adlam et al., 2006; Murphy et al., 2006). In this particular population, the impaired performance in CFT has also been reported to be predictive of subsequent progression to dementia (Amieva et al., 2004; Tierney et al., 2005; Hodges et al., 2006; Caroli et al., 2007; Maioli et al., 2007; Aretouli et al., 2011; Hanseeuw and Ivanoiu, 2011; Molinuevo et al., 2011; Blanco Martin et al., 2016; Gallucci et al., 2018), even though some studies have failed to replicate this finding (Alegret et al., 2014; Callahan et al., 2015; Kim et al., 2017; Russo et al., 2017). In more recent years, the performances of individuals with DAT or MCI on CFT have been assessed, taking into account several psycholinguistic variables, modifications in which may reflect a subtle impairment of the lexical-semantic system. Among the variables that could influence the results obtained on CFTs, age of acquisition, frequency of use, and typicality of words have received particular attention. In fact, there is evidence that in normal individuals, lexical retrieval is facilitated for words acquired early in life, that correspond to typical representatives of a specific category, and are frequently present in speech (Schilling et al., 1998; Juhasz, 2005; Holmes and Ellis, 2006). Individuals with DAT have been reported to produce words with higher frequency, lower age of acquisition, and higher typicality than healthy individuals (Sailor et al., 2004, 2011; Forbes-McKay et al., 2005; Venneri et al., 2008). We have previously reported that individuals with MCI display an increased level of typicality of words produced in a CFT test, and that individuals producing highly typical words are more prone to developing dementia during the follow-up period (Vita et al., 2014).

**FIGURE 1:**
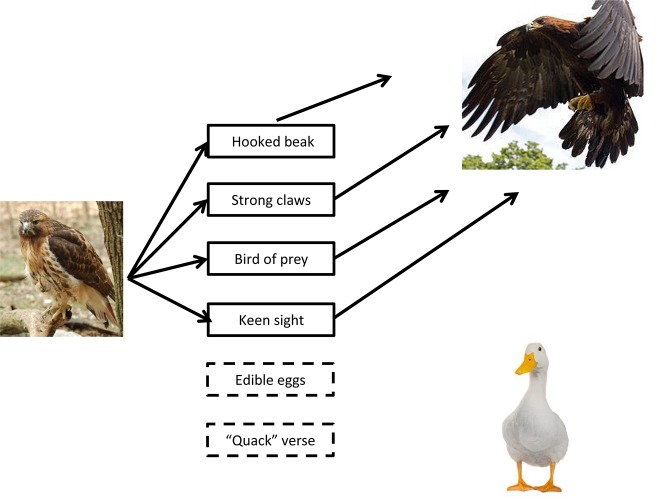
In category fluency tasks, the production of a subsequent word may be influenced by the previous one through the activation of shared features that may facilitate the retrieval of the following lexical entry. In the example depicted here, the production of the word “hawk” may cause the activation of features such as the ones marked with a continuous line that are shared with “eagle,” but not with “duck.” Thus, “eagle” has a higher probability of being produced, than “duck.” When this kind of strategy (resembling classical “clustering”) is exhausted, the subject must “switch” to other subcategories.

The performance in categorical fluency test in DAT and MCI has been variously attributed to an impairment of semantic memory or a reduced ability to access the verbal representation of otherwise intact conceptual representations (Foster et al., 2013; Salehi et al., 2017; Tchakoute et al., 2017). The investigation of the relationships between words generated during verbal fluency tasks may be of some usefulness in trying to clarify this issue. In fact, it is predictable that items produced during a CFT should be connected by a shared meaning (such as, membership in the same category). Thus, in an intact semantic system the activation of successive lexical entries will be easier for concepts that are close to the previous words in terms of meaning (or of shared taxonomic, perceptual or functional features). For example, in the case of birds, one may predict that after “hawk” it is more probable to produce the word “eagle” than the word “duck,” since the former shares more features with the word “hawk” (bird of prey with hooked beak and strong claws) than the latter (see Figure 1). This prediction is grounded on models of semantic memory in which concepts are represented by nodes connected by meaningful relationships (Collins and Loftus, 1975; Levelt et al., 1999; Levelt, 2001; Voorspoels et al., 2014) whose activation precedes lexical access (Levelt et al., 1999; Levelt, 2001). If semantic memory is impaired, it is possible to predict a deviation from this process, which may lead to a different distribution of measures of semantic relations within patients.

Semantic relations may be the principal contributor to the phenomenon of “clustering,” that is the tendency to produce clusters of words that belong to the same subcategory (for example, “birds of prey”). This phenomenon can be contrasted with the transition to a different subgroup of words, which is called “switching.” The general claim is that clustering relies upon the integrity of semantic memory whereas switching is related to executive functions (Troyer et al., 1997, 1998). Such phenomena have been extensively investigated in several clinical conditions, including schizophrenia (Aloia et al., 1996; Moelter et al., 2005; Voorspoels et al., 2014), bipolar disorders (Chang et al., 2011) and DAT, in which the generation of fewer clusters and switches than healthy individuals has been described (Fagundo et al., 2008; Haugrud et al., 2011). The main approaches to the clustering/switching issue can be summarized into two general principles (Voorspoels et al., 2014). The first is based on the observation that individuals tend to cluster similar exemplars of the category in their response order. The strength of the relation between two items derives from the level of similarity between them. In studies based on this principle, the level of the relation between two words is derived from the number of words that separate the two target lexical entries in the sequence (Prescott et al., 2006). The second approach is based on the frequency of co-occurrence of words in the response sequence, and represent an application of Single Value Decomposition (Sung et al., 2012). Both of these approaches have yielded significant results about distortions of semantic representations in clinical populations. However, recent evidence (Voorspoels et al., 2014) suggests that such methodologies may not yield consistent and reliable results in comparisons between healthy participants (HP) and individuals with clinical conditions. The authors performed repeated sampling from a group of patients and controls, and showed that the similarity scores obtained from the two approaches vary greatly across samples of the same population, for both patients and controls (Voorspoels et al., 2014). Thus, it may be useful to explore the use of sample independent measures to re-assess clustering in CFT.

**FIGURE 2:**
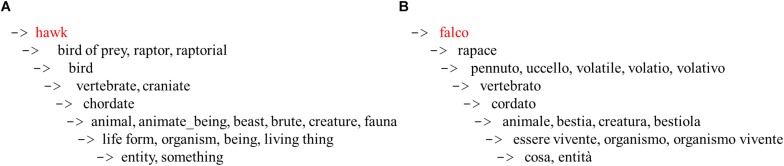
**(A,B)** Complete hierarchical trees for the word “hawk” and the corresponding Italian term “falco,” as derived from WordNet and MultiWordNet.

In recent years, several lexical databases have been developed, collecting and systematizing lexical entries on the basis of semantic hierarchies. The WordNet database (Fellbaum, 1998, 2005) is a large English lexical database. It is organized in a hierarchical fashion, in which lexical and conceptual links connect words and groups of words. The general structure of Wordnet is based on semantic relationships among lexical entries more than on formal attributes of words (such as those found in thesauruses). The basic element of WordNet is the *synset* (set of synonyms). Each synset is defined by a *gloss*, that is a verbal description of the concept to which it refers. Several conceptual relationships are taken into account in WordNet. Nouns, which is the grammatical class involved in the present study, are organized according to hypernym, hyponym, meronym, and holonym relations. Hypernym and hyponym refer to the taxonomic (“is-a-kind-of”) relationship (if “eagle” is a kind of “bird,” then “eagle” is a hyponym of “bird” and “bird” is a hypernym of “eagle”). Meronym and holonym are connected with the “is-a-part-of” relationship (if “beak” is a part of “eagle,” then “beak” is a meronym of “eagle,” and “eagle” is a holonym of “beak”). Each synset is embedded in an ordered hierarchy, based on the “is a” relationship (Fellbaum, 2005), resembling the categorical organization of conceptual knowledge. Figure 2A displays the complete hierarchy (“hypernym”) of the synset “hawk” as derived from WordNet. The structure of the Wordnet database makes it suitable for the assessment of semantic relationships between different lexical entries.

Semantic relations may be taken into account from at least two points of view (Pedersen et al., 2004). In fact, two concepts may be associated on the grounds of similarity, that is, how much two concepts are alike. In this case, measures of semantic relations are based on the taxonomic structure of the database. Semantic relations could also represent the effect of not exclusively taxonomic relationships (for example, “it is opposite to”); this is the case, according to Pedersen et al. (2004) of semantic relatedness. It would be interesting to investigate both these aspects of semantic relations in clinical populations; in fact, it is conceivable that the effect of an impairment of the semantic system may involve its taxonomic organization (that could be explored by measures of semantic similarity) or more general relationships between lexical entries (explored by measures of semantic relatedness).

The aims of the present study were: (a) to compare measures of semantic relations obtained from a CFT between HP and individuals with an amnesic form of MCI (aMCI), in particular in patients who progressed to dementia, and (b) to verify the possibility of identifying word clusters using measures of semantic relations obtained from Wordnet, and to compare the number of such clusters observed in HP and individuals with aMCI.

## Materials and Methods

### Subjects

The study sample of individuals with aMCI was consecutively enrolled from among persons referring to the Memory Clinic of the Policlinico Universitario “A. Gemelli” in Rome. The diagnosis of MCI was formulated according to current clinical criteria (Winblad et al., 2004; American Psychiatric Association, 2013). For the definition of aMCI the following characteristics where considered: (1) memory disturbances reported by the subject or an informed caregiver, with the specification that such disturbances represented an impairment as compared to past time; (2) evidence of an objective memory deficit as measured by means of Rey Auditory Verbal Learning Test (RAVLT – delayed recall) and delayed recall of the Rey–Osterrieth’s Complex Figure; the patients must perform below the cut-off score on at least one of the tests to be diagnosed with aMCI; and (3) preserved efficiency in activities of daily life: the patient must be able to carry on activities as usual or with minimal difficulty (for example, slight slowing).

The cut-off scores were obtained from the norms for the Italian population published in respective standardization papers for RAVLT (Carlesimo et al., 1996) and Rey–Osterrieth’s Complex Figure copy and delayed recall (Caffarra et al., 2002); the cut-off scores, corresponding to the 5th percentile, and after correction for age, education, and gender, were 4.69 for RAVLT, and 9.46 for delayed recall of Rey–Osterrieth’s Complex figure. The assessment of efficiency in activities of daily living was based on narrative reports and the administration of the Activities of Daily Living (Katz et al., 1963) and Instrumental Activities of Daily Living (Lawton and Brody, 1969) scales to the main informant (generally the spouse or children). All of the enrolled individuals obtained a Clinical Dementia Rating scale score of 0.5.

Exclusion criteria were previous or concomitant neurological or psychiatric disturbances documented by the neurologist who diagnosed aMCI (DQ or CM); further exclusion criteria were concomitant medical conditions that could cause or influence cognitive impairment (such as, but not limited to: liver or renal failure; vitamin B12 deficiency; hypothyroidism; etc.). None of the patients was receiving psychotropic drugs. Non-native Italian speakers were also excluded.

The group of HP was enrolled from among caregivers (mainly spouses) of persons referring to our Memory Clinic. All of them underwent the Mini Mental State Examination (Magni et al., 1996) and had to have a score above 27, and a clinical evaluation to assess the presence of the same exclusion criteria, as applied to individuals with aMCI (see above).

We obtain informed consent from all persons accessing medical services at the clinic and ask them permission for the use of their clinical data for research purposes. Data pertaining to HP were obtained from the previously published standardization study on the CFT used in the present study (Quaranta et al., 2016).

### Follow-Up

Individuals with aMCI were regularly followed up and subjected to a comprehensive neuropsychological evaluation every 6 months to assess progression to dementia. A diagnosis of dementia was formulated when current clinical criteria (American Psychiatric Association, 2013) were satisfied; in particular, the subject should have displayed an impaired ability to autonomously perform activities of daily life and obtained a CDR score equal to or higher than 1.

### Semantic Verbal Fluency Test

The participants were examined by means of a CFT using the semantic categories “birds” and “pieces of furniture” (Quaranta et al., 2016); they were given one minute each to recall as many nouns as possible belonging to the two categories (assessed separately). The output was recorded and then written down following the order of production.

### Semantic Relations

The strength of the semantic relations was determined for lexical entries that were produced consecutively (word1–word2: value1; word2–word3: value2, etc.). Thus, each single word entered the analysis twice (with the exception of the first and last ones); repetitions and errors (words not belonging to the category) were not taken into account in the analysis. Specifically, if a repetition occurred, the corresponding word was taken into account only at its first appearance.

Values for each word pair were obtained from Wordnet (Fellbaum, 1998). In the present study, two measures were taken into account: the first one was Path Length, a similarity measure corresponding to the inverse of the shortest path length connecting two concepts through the taxonomy, the length of the path being the number of nodes connecting two concepts; for example, if we consider the words “hawk” and “eagle,” the shortest path is “hawk”-“bird of prey”-“eagle,” that is 3; thus the Path length measure will be 0.333. The inverse of the shortest path length between two lexical entries was chosen for clarity. In fact, path length is inversely proportional to the similarity of the words, whereas higher inverse path length indicates higher similarity, and vice-versa. The second measure was Extended Gloss Overlap (Banerjee and Pedersen, 2003), a relatedness measures that takes into account the amount of overlap between the glosses defining two different concepts. This measure was developed to explore not only the overlaps between the glosses defining the two concepts taken into account, but also between the glosses related to their hypernym, hyponym, meronym, and holonym. Since it is based on the verbal description of the synsets, the latter measure is significantly influenced by the shared features of two concepts.

Path length and Extended Gloss Overlap measures were obtained using WordNet: Similarity (Pedersen et al., 2004), a freely available software package that takes as input two concepts, and gives as output a numeric value that represents the degree to which the concepts are similar or related. Since the output of the verbal fluency test was in Italian, the words needed to be translated to be processed in WordNet: Similarity. The translation was carried out referring to MultiWordNet (Pianta et al., 2002), a multilingual lexical database in which the taxonomy of WordNet is applied to other languages, including Italian, Spanish, Portuguese, Hebrew, Romanian, and Latin. The lexical entries were searched for on the MultiWordNet online interface and the correct definition (for example: “hawk: diurnal bird of prey typically having short rounded wings and a long tail”) was selected among the ones provided by WordNet to obtain the Semantic Similarity and Relatedness measures. Figures 2A,B display the hypernym of the word “hawk” and the corresponding Italian term (“falco”). As shown, the taxonomic position of both words was overlapping.

Measures derived from artificial lexical databases have been demonstrated to have a fair to good correlation with human judgment on semantic relatedness (Cramer, 2008). However, the validity of measures derived automatically from databases has been generally assessed on restricted sets of words. For this reason, after completing the collection of data from the aMCI and HP samples, we collected data on the human judgment of semantic relatedness for all of the word pairs recorded. Data were gathered from 30 age- and literacy-matched adults, recruited from among the caregivers of patients referred to our Neuropsychology Service, and who agreed to participate in the study. For each word pair presented in Italian, the participants were asked to score how much they were related or similar to one another (“how much do you think that these two words are related to each other?”). Responses were taken into account only when the subject knew both words in the word pair. Individual responses ranged from 1 (not related) to 10 (highly related). For each word pair, the mean value of Human Estimation (HE) of semantic relations was computed.

### Clustering

The measures of semantic relations were used to explore the generation of clusters of words while performing the CFT. The analysis was performed by identifying the number of clusters produced by each subject considering the WordNet measures. Groups of words that were characterized by both Path length and Extended Gloss Overlap greater than the mean value of the measures by each individual, were considered as clusters. The clusters were coded manually and the number of clusters for each subject was taken into account. The minimum number of words needed to produce a cluster was three. In fact, if the cluster could be formed by only two words, linked by measures of semantic relations higher than the average value for each individual, an artificially high number of clusters could be detected in subjects who produced more words. Furthermore, each word could participate in the formation of only one cluster.

### Statistics

The statistical analyses were performed on R statistical package. Comparisons between means of continuous variables were carried out by means of the *t*-test, with Levene’s test for equality of variance, or the one-way ANOVA, with Tukey’s test for *post hoc* comparisons. Correlations between variables were examined using Pearson’s correlation coefficient (r). The predictive effect of the measures of semantic relations on the CFT total score was assessed by means of linear regression analysis. Given the conceivable differences of raw score (number of produced items) obtained on the CFT between HP and aMCI, the measures were weighted for the number of words by multiplying the values referred to a single individual by the number of words the same individual produced.

## Results

### Sample Characteristics

The sample comprised of 34 individuals (18 females) with aMCI (mean age: 73.1 ± 4.30; mean education: 9.6 ± 5.48; mean MMSE score: 26.1 ± 2.05) and 29 HP (15 females; mean age: 72.5 ± 4.59; mean education: 10.0 ± 3.61; mean MMSE score: 29.2 ± 0.90). The two groups did not differ in terms of age (|*t*| = 0.591; *p* = 0.557) and education (|*t*| = 0.368; *p* = 0.715) but, as expected, the mean score obtained by individuals with aMCI on MMSE was significantly lower than that obtained by HP (|*t*| = 7.386; *p* < 0.001). No difference was found in gender distribution (χ2 = 0.009; *p* = 0.923).

At the 3-year follow-up, progression to dementia was observed in 10 patients (29.4% of the initial sample). In all these cases, DAT was diagnosed.

### Performance on the Categorical Verbal Fluency Test

The number of word pairs analyzed were 498 (birds: 262; pieces of furniture: 236) in HP, and 395 (birds: 174; pieces of furniture: 221) in aMCI. Table 1 reports the mean performance of HP and aMCI individuals on the CFT. HP obtained higher scores than both aMCI who converted to dementia (aMCI-conv) and aMCI who remained stable (aMCI-stable).

**TABLE 1:** Mean values of the number of lexical entries produced, path length and extended gloss overlap for the “birds” and “pieces of furniture” categories.

	**HP**		**aMCI-stable**		**aMCI-conv**		***F***	***p***	**η^2^**
	**Mean**	***SD***	**Mean**	***SD***	**Mean**	***SD***			
Birds	10.3^a,b^	3.33	6.3^a^	2.54	5.5^b^	1.58	14.585	<0.001	0.314
Pieces of furniture	8.6^a,b^	2.03	7.1^a^	1.75	6.7^b^	2.46	6.637	0.002	0.181
**Path length**									
Birds	0.189	0.0255	0.193	0.0488	0.181	0.0374	1.666	0.190	0.008
Pieces of furniture	0.262^a^	0.0398	0.260	0.0355	0.249^a^	0.0322	3.112	0.049	0.030
**Ext. gloss overlap**									
Birds	24.963^a^	11.9059	22.498	16.9829	18.102^a^	10.2380	5.630	0.004	0.027
Pieces of furniture	35.360^a^	14.2137	34.170	14.5907	29.401^a^	13.7169	4.107	0.017	0.031

*HP, healthy participants; aMCI-stable, persons with aMCI who remained stable; aMCI-conv, persons with aMCI who converted to dementia. ^*a*^HP vs. aMCI-stable *p* < 0.05. ^*b*^HP vs. aMCI-conv *p* < 0.05.*

### Relationship Between Path Length, Extended Gloss Overlap and Human Estimation

The group of participants who provided the estimation of semantic relations between word pairs was formed by 30 persons (15 female) with a mean age of 72.9 years (*SD* = 4.21) and a mean education of 9.9 years (*SD* = 4.72); a one-way ANOVA showed no significant difference in age (*F* = 0.151; *p* = 0.889) and education (*F* = 0.064; *p* = 0.938) in comparison with HP and individuals with aMCI.

The analysis of the correlation between Path Length and Extended Gloss Overlap measures was carried out using the words produced by the entire sample and then repeated using only the words produced by HP.

When carried out on the whole sample of words, the correlations of HE with Path Length (*r* = 0.428; *p* < 0.001), and with Extended Gloss Overlap (*r* = 0.367; *p* < 0.001) were quite fair. When only words produced by HP were taken into account, the results were similar for both Path Length (*r* = 0.469; *p* < 0.001) and Extended Gloss Overlap (*r* = 0.364; *p* < 0.001).

### Path Length

**GRAPH 1:**
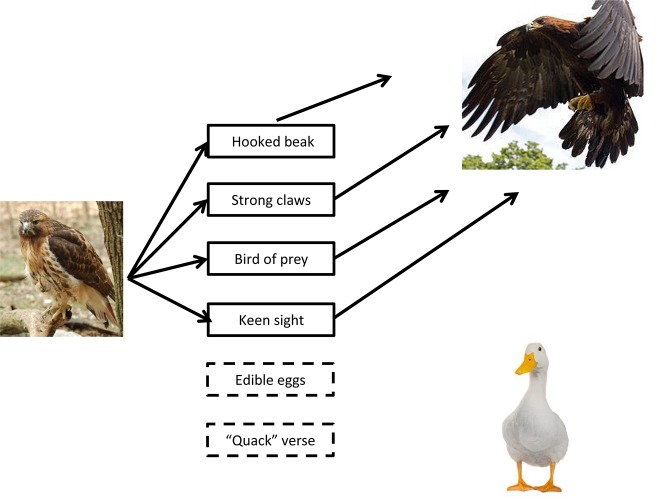
Mean values of path length in the “birds” and “pieces of furniture” categories among healthy participants (HP), aMCI who remained stable (aMCI-stable) and aMCI who converted to dementia (aMCI-conv). Error bars correspond to 95% Confidence Interval. ^∗^HP vs. aMCI-conv *p* < 0.05.

For the “birds” category, an ANOVA comparing the path length scores among groups (HP, aMCI-stable, a-MCI-conv) showed no significant differences (*F* = 1.666; *p* = 0.190; Table 1 and Graph 1).

For the “pieces of furniture” category, the ANOVA showed an effect of group (*F* = 3.112; *p* = 0.049). The *post hoc* tests showed a difference between HP and aMCI-conv (*p* = 0.035) but no differences between aMCI-stable and HP, nor between aMCI-stable and aMCI-conv (Table 1 and Graph 1).

### Extended Gloss Overlap

**GRAPH 2:**
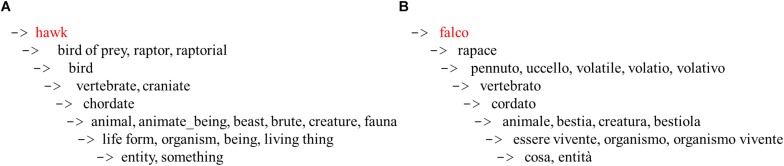
Mean values of extended gloss overlap in the “birds” and “pieces of furniture” categories among HP, aMCI who remained stable (aMCI-stable) and aMCI who converted to dementia (aMCI-conv). Error bars correspond to 95% Confidence Interval. ^∗^HP vs. aMCI-conv *p* < 0.05.

For the “birds” category, an ANOVA comparing the path length scores among groups (HP, aMCI-stable, a-MCI-conv) showed a significant difference (*F* = 5.630; *p* = 0.004); the *post hoc* analysis showed a significant difference between HP and aMCI-conv (*p* = 0.003; Table 1 and Graph 2).

For the “pieces of furniture” category, the ANOVA showed a significant effect of group (*F* = 4.107; *p* = 0.017); the *post hoc* test showed a significant difference between aMCI-conv and HP (*p* = 0.049) and aMCI-conv and aMCI-stable (*p* = 0.001; Table 1 and Graph 2).

### Effect of Path Length and Extended Gloss Overlap on the Categorical Verbal Fluency Test Performance

A linear regression analysis, controlled for age, literacy, and gender, showed that the path length scores did not explain the variance in total scores either for the “birds” (*B* = 0.400; *p* = 0.969) or the “pieces of furniture” (*B* = 7.96; *p* = 0.101) categories. Extended Gloss Overlap showed a significant effect of the performance for the “birds” category (*B* = 0.062; *p* = 0.043), but not for the “pieces of furniture” category (*B* = 0.012; *p* = 0.366).

### Clustering

**GRAPH 3:**
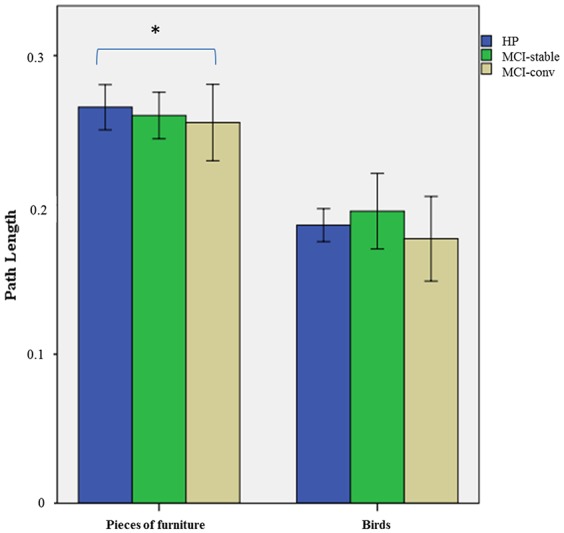
Mean number of clusters identified for the “birds” and “pieces of furniture” categories among HP, aMCI who remained stable (aMCI-stable) and aMCI who converted to dementia (aMCI-conv). Error bars correspond to 95% Confidence Interval. ^∗^HP vs. aMCI-conv *p* < 0.05. #HP vs. aMCI-stable *p* < 0.05.

When the “birds” category was taken into account, the ANOVA showed a significant effect of group (*F* = 66.045; *p* < 0.001); the *post hoc* analysis showed a statistically significant difference between HP and aMCI-stable (*p* < 0.001) and aMCI-conv (*p* < 0.001), whereas no difference was found between aMCI-stable and aMCI-conv (*p* = 0.250) (Table 2 and Graph 3). The same results were observed when the “pieces of furniture” category was taken into account, with HP forming more clusters than both aMCI-stable and aMCI-conv (*p* < 0.001 for both), and no differences between aMCI-stable and aMCI-conv (*p* = 0.993) (Table 2 and Graph 3).

**TABLE 2:** Comparison of mean number of clusters identified according to measures of semantic relations.

	**HP**		**aMCI-stable**		**aMCI-conv**		***F***	***p***
	**Mean**	***SD***	**Mean**	***SD***	**Mean**	***SD***		
Birds	1.90^a,b^	0.733	1.15^a^	0.383	1.32^b^	0.471	66.045	<0.001
Pieces of furniture	2.24^a,b^	0.838	1.83^a^	0.777	1.81^b^	0.656	15.759	<0.001

*HP, healthy participants; aMCI-stable, persons with aMCI who remained stable; aMCI-conv, persons with aMCI who converted to dementia. ^*a*^HP vs. aMCI-conv *p* < 0.05. ^*b*^HP vs. aMCI-stable *p* < 0.05.*

## Discussion

In the present study, we explored the hypothesis that the level of semantic similarity and/or relatedness between words produced by individuals with aMCI could differ compared to HP, and that this difference could be more pronounced in patients who progressed to dementia during follow-up.

The individuals with aMCI who eventually converted to dementia during the follow-up period displayed a reduced level of mean strength of semantic relations for both the “birds” and “pieces of furniture” categories when a measure of semantic relatedness (extended gloss overlap) was taken into account and, to a lesser extent, and only for “pieces of furniture,” when the semantic similarity measure (path length) was considered.

This finding may represent the effect of impaired semantic memory. In fact, the reduction of the strength of semantic relationships between consecutive terms produced in the CFT might reflect the weakening of the links between concepts, which in turn might represent the amount of shared features between concepts (Kiefer and Pulvermüller, 2012). In this way, the influence of the previous words on the subsequent ones, which might be related to the activation of common features (Figure 1), could weaken, and thus, words with lower levels of similarity and/or relatedness might be produced, leading to a general reduction in the strength of semantic relations. However, the effect size of the differences observed in our sample are rather small, and deserves further comment. In fact, the organization of WordNet resembles the structure of classical models of semantic memory, in which knowledge of concepts is stored in interlinked nodes that are rather stabile (Collins and Loftus, 1975). Recent evidence suggests that concepts may be represented in a distributed and flexible way that is influenced by the semantic context and previous individual experience (Kiefer and Pulvermüller, 2012). Thus, it is possible that the measures used in the present paper did not reveal the total variability between HP and individuals with aMCI, since individual factors and semantic context may model the conceptual representations.

The results obtained from the analysis of clustering are in keeping with previous works (Chan et al., 1993; Troyer et al., 1998; Fagundo et al., 2008; Haugrud et al., 2011) and corroborate the hypothesis of an impairment of semantic memory very early in the course of DAT. Previous works have reported that individuals with DAT or MCI showed an impaired ability to form clusters while performing a CFT, in line with the prediction of impaired verbal semantics in DAT. However, most previous studies were based on the *a priori* definition of subcategories or on the extraction of subcategories from the performances of normal individuals (Chan et al., 1993; Troyer et al., 1997, 1998; Sung et al., 2012; Zhao et al., 2013; Voorspoels et al., 2014). Our results showed that individuals with aMCI produced a lower number of clusters when compared to HP, with no significant difference between aMCI patients who progressed to dementia and those who remained stable.

It is of some interest to point out that the extended gloss overlap was reliable in detecting differences among the groups for both categories, whereas path length only detected some difference in the “pieces of furniture” category. This finding could be due to the specific characteristics of the measures; path length is a semantic similarity measure (Pedersen et al., 2004), that is based on the taxonomic structure of the lexical database. On the other hand, extended gloss overlap is a semantic relatedness measure, that takes into account non-taxonomic relationships, and is based on the overlap between definitions (glosses) of words (Banerjee and Pedersen, 2003). Being somehow broader than path length in estimating semantic relations, extended gloss overlap could be more reliable in detecting an impairment of the semantic system. The same reason could explain the fact that only extended gloss overlap predicted to some extent the performance on the CFT (though limited to the “birds” category). Furthermore, extended gloss overlap is based on shared features (as described in the glosses) that could be more relevant for birds than for articles of furniture during word generation. However, we did not investigate this specific effect, and thus, it is not possible to draw definite conclusions on this issue.

Our results may strengthen the hypothesis of a very early impairment of semantic memory in Alzheimer’s disease (Venneri et al., 2018). In fact, semantic memory is a component of declarative memory, organized on the basis of context shared by related lexical items (Kiefer and Pulvermüller, 2012), and involves the knowledge of concepts and words. The neuroanatomical substrate of such a component of long term memory is a matter of debate, given the great complexity of semantic representations, and accordingly, of the neural structures connected with it (Huth et al., 2016). There is general agreement that the medial temporal lobe structures are involved in both episodic and semantic memory. In particular, a key role in semantic memory has been attributed to sub-hippocampal structures, and in particular to the perirhinal cortex (Didic et al., 2011), whereas the proper hippocampus is mainly connected to episodic (context-rich) memory. The perirhinal and entorhinal cortices are affected very early by tau pathology in the transentorhinal stage of Alzheimer’s disease (Braak and Braak, 1991), even before the involvement of the hippocampus (limbic stage). Individuals with MCI may show some degree of impairment in semantic memory that could be detected through a fine-grained analysis of their performance on language tests. Further investigations on the relationship between semantic memory and their putative neuroanatomical substrates are warranted.

The main limitation of this study is that the semantic measures were obtained from a lexical database of a foreign language (English vs. Italian), and thus, our results require cautious interpretation. In fact, even though significant, the correlation between the measures obtained from WordNet: Similarity and Human Estimation was not very strong. This finding could be due to the fact that the level of similarity of two words as judged by individuals who were requested to provide an estimation, is quite different from the precise measurement provided by a systematic database constructed according to a specific taxonomic structure. However, previous observations have shown good cross-cultural reliability of the verbal fluency test for the “birds” and “pieces of furniture” categories in terms of typicality of the words produced (Quaranta et al., 2016), thus supporting a fair overlap among at least some aspects of semantic representation of such categories with English speakers. Furthermore, the translation was performed using the MultiWordNet database (Pianta et al., 2002), that allows the direct comparisons of the glosses obtained from WordNet with the Italian definitions of each lexical entry, and that share the same taxonomic organization as WordNet.

Another potential limitation of the present study is that the HP who underwent the CFT and individuals who were asked to provide an estimation of semantic relations between couples of words did not undergo a thorough neuropsychological examination, and thus, the diagnosis of subtle cognitive impairment cannot be completely ruled out. Finally, the number of individuals with aMCI who participate in the study was quite small.

## Conclusion

In conclusion, measures of semantic relations could be useful in the assessment of MCI as a possible marker of very early impairment of semantic memory and as a possible predictor of progression to dementia.

## Data Availability Statement

The datasets generated for this study are available on request to the corresponding author.

## Ethics Statement

Ethical review and approval was not required for the study on human participants in accordance with the local legislation and institutional requirements. The patients/participants provided their written informed consent to participate in this study.

## Author Contributions

DQ, CP, and GG contributed conception and design of the study. AC and AM collected data from patients and organized the database. DQ, CP, and AC performed the statistical analysis. DQ wrote the first draft of the manuscript. CM, CP, and GG wrote sections of the manuscript. All authors contributed to the manuscript revision, and read and approved the submitted version.

## Conflict of Interest

The authors declare that the research was conducted in the absence of any commercial or financial relationships that could be construed as a potential conflict of interest.
